# Different properties of ACPA and IgM-RF derived from a large dataset: further evidence of two distinct autoantibody systems

**DOI:** 10.1186/ar2704

**Published:** 2009-05-21

**Authors:** Jennie Ursum, Wouter H Bos, Rob J van de Stadt, Ben AC Dijkmans, Dirkjan van Schaardenburg

**Affiliations:** 1 Jan van Breemen Institute, Dr. Jan van Breemenstraat 2, 1056 AB Amsterdam, The Netherlands; 2 VU University Medical Centre, Postbus 7057, 1007 MB Amsterdam, The Netherlands

## Abstract

**Introduction:**

The aim of this study was to examine seroconversion and the relationship with age and inflammation of autoantibodies in a large group of patients attending an outpatient rheumatology clinic.

**Methods:**

Levels of antibodies to citrullinated proteins/peptides (ACPAs) and IgM rheumatoid factor (IgM-RF) were determined in 22,427 samples collected from 18,658 patients. The diagnosis was derived from a diagnosis registration system. The degree of seroconversion in repeated samples and the correlation of levels with age and inflammatory markers were determined for ACPA and IgM-RF in rheumatoid arthritis (RA) and non-RA patients.

**Results:**

Seventy-one percent of RA patients (n = 1,524) were ACPA-positive and 53% were IgM-RF-positive; in non-RA patients (n = 2,245), the corresponding values were 2% and 4%, respectively. In patients with at least two samples (n = 3,769), ACPA status was more stable than IgM-RF status in RA patients. ACPA- or IgM-RF-negative non-RA patients seldom became positive. ACPA positivity was unrelated to age in both RA and non-RA patients. IgM-RF positivity was unrelated to age in RA patients; however, it increased with age in non-RA patients. The correlation between autoantibody levels and inflammatory markers was low in general and was somewhat higher for IgM-RF than for ACPA.

**Conclusions:**

ACPA status is more stable in time and with increasing age than IgM-RF status, further establishing its role as a disease-specific marker. ACPA and IgM-RF levels are only moderately correlated with markers of inflammation.

## Introduction

One of the frequent characteristics of rheumatoid arthritis (RA) is the presence of antibodies to citrullinated proteins/peptides (ACPAs) and/or IgM rheumatoid factor (IgM-RF) [1]. IgM-RF targets the Fc fragment of IgG and is observed in about 60% to 65% of RA patients, but it is also frequently observed in other inflammatory diseases [2,3]. ACPAs comprise a group of antibodies that are highly specific for RA: among those are antibodies against cyclic citrullinated peptide (CCP) [4]. ACPAs target citrullinated proteins and are observed in around 70% of RA patients. In contrast to IgM-RF, ACPA is highly specific for RA (specificity 80% versus 96%, respectively) [3].

Besides their well-established superior specificity for RA, several other properties of ACPA are distinct from IgM-RF. About 50% to 70% of early-RA patients are ACPA-positive, and this phenotype remains fairly stable thereafter [2,5,6], even during treatment with tumour necrosis factor (TNF)-blocking agents [7]. On the other hand, IgM-RF levels decrease during antirheumatic treatment [8] and 17% of IgM-RF-positive RA patients turned negative after 6 months of anti-TNF treatment [9].

Furthermore, IgM-RF [10], but not ACPA [11], is sometimes present in healthy older persons, suggesting that RF can be a consequence of nonspecific immune activation. Moreover, it has been suggested that IgM-RF production also is a consequence of the rheumatoid inflammation whereas ACPA may have pathophysiological properties. Evidence supporting this concept is emerging [12]. For instance, ACPA precedes IgM-RF in the preclinical phase [13] and the change in IgM-RF levels during anti-TNF treatment is associated with the change in acute-phase response; this is not observed for ACPA [9]. These data suggest that ACPA and IgM-RF represent two different autoantibody systems. ACPAs are disease-specific, their presence is fairly stable in time and does not increase with age, and ACPA levels are not correlated with the acute-phase response. On the other hand, IgM-RF is less disease-specific, its presence increases with age in healthy/non-RA individuals, and its levels are correlated with the acute-phase response.

Most of these data have emerged from studies of selected populations with small sample sizes. In the present study, we sought to confirm the stability of ACPA in time, the increased IgM-RF frequency with age, and the correlation of IgM-RF with the acute-phase response using a repository of over 22,000 serum samples collected from over 18,000 patients attending a rheumatology clinic network in The Netherlands.

## Materials and methods

ACPA and IgM-RF levels were determined in 22,427 samples, which were collected from 18,658 patients between August 2003 and August 2007. These patients attended one of the outpatient rheumatology clinics of the Jan van Breemen Institute in the Amsterdam region of The Netherlands. Each patient's final diagnosis was obtained from the International Classification of Diseases version 10 diagnosis registration system, which reflects the opinion of the treating rheumatologist. The diagnosis was categorized into five groups according to the following codes: RA, polyarthritis or oligoarthritis, spondylarthropathy (including ankylosing spondylitis, reactive arthritis, psoriatic arthritis, arthritis associated with inflammatory bowel disease, and undifferentiated spondyloarthropathy), osteoarthritis, and other (including arthralgia, fibromyalgia, and no final diagnosis). The latter four groups were also combined and classified as 'non-RA'. The disease duration at the time of autoantibody testing was variable and unknown. For the association between age and autoantibody positivity, patients were grouped according to their age at the first available sample: younger than 30, 30 to 39, 40 to 49, 50 to 59, 60 to 69, 70 to 79, and 80 years old or older. The local ethics committee approved the study protocol and waived the need for informed patient consent.

### Laboratory investigations

All measurements were routinely performed at the certified clinical laboratory of the Jan van Breemen Institute. After the first sample, sequential samples were obtained as a part of routine or protocollar care. In the case of routine care, samples were obtained at the request of the rheumatologist at a nonspecific time point. In the case of protocollar care, samples were obtained annually. ACPA levels were determined by second-generation anti-CCP enzyme-linked immunosorbent assay (ELISA) (Axis-Shield, Dundee, UK). Sera reaching 1,000 arbitrary units (AU) were not further diluted. The cutoff level for ACPA positivity was set at 5 AU/mL in accordance with the instructions of the manufacturer. IgM-RF levels were determined by an in-house ELISA. The cutoff level for IgM-RF positivity was set at 30 international units (IU)/mL, which was determined on the basis of receiver operating characteristic curves described previously [14]. Erythrocyte sedimentation rate (ESR) was measured according to the Westergren method using a Starrsed analyser (Mechatronics, Zwaag, The Netherlands), and the reference value was less than 15 mm per first hour. C-reactive protein (CRP) was measured on a Cobas 6000 analyser (Roche, Woerden, The Netherlands) in accordance with the instructions of the manufacturer, and the reference value was less than 10 mg/L.

### Analysis

The effect of age on ACPA and IgM-RF positivity was determined using the chi-square test. Correlations between levels of antibodies and levels of markers of inflammation were determined with Spearman correlation. All analyses were performed using SPSS 16.0 software (SPSS Inc., Chicago, IL, USA).

## Results

Serum samples were available in 18,658 patients: 3,116 patients with RA, 1,063 with polyarthritis or oligoarthritis, 818 with spondylarthropathy, 2,736 with osteoarthritis, and 10,925 classified as other. A second sample was available in 1,524 patients with RA, 419 with polyarthritis or oligoarthritis, 195 with spondylarthropathy, 333 with osteoarthritis, and 1,298 classified as other. Of all second samples, 35% were obtained annually as part of a protocol, and the others were obtained according to physician request. These 35% can be divided into patients receiving routine care (24%), described elsewhere [15], and those receiving anti-TNF treatment (11%). In the first sample of the 18,658 patients, the percentages of patients with positive ACPA were 71% in the RA group and 2% in the non-RA group. Rates of IgM-RF positivity were 53% in the RA group and 4% in the non-RA group. In the second sample (n = 3,769), the percentages of patients with positive ACPA were 70% in the RA group and 7% in the non-RA group. Rates of IgM-RF positivity were 49% in the RA group and 10% in the non-RA group.

### Switch in antibody to citrullinated proteins/peptides or IgM rheumatoid factor status between first and second samples

**Figure 1:**
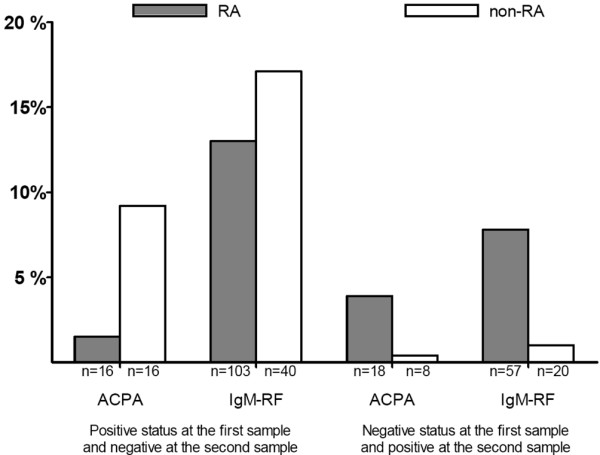
Percentage of rheumatoid arthritis (RA) and non-RA patients with a change in positivity in antibodies to citrullinated proteins/peptides (ACPA) and IgM rheumatoid factor (IgM-RF) between the first and second samples.

In patients with at least two samples, the stability of the autoantibody status was assessed (n = 3,769). The median times between the first and second samples were similar for RA patients (n = 1,524) and non-RA patients (n = 2,245): 11 months (interquartile range [IQR] 4 to 13 months) and 9 months (IQR 3 to 16 months), respectively. In RA patients, the percentages of patients switching from ACPA positivity to negativity and from ACPA negativity to positivity were lower compared with percentage changes in IgM-RF. In initially ACPA-positive RA patients, 1% of the second sample was negative, whereas 13% of the second sample in IgM-RF-positive RA was negative (*P *< 0.001). In initially ACPA-negative RA patients, 4% of the second sample was ACPA-positive, whereas 8% of the initially IgM-RF-negative RA patients became positive (*P *< 0.001). Furthermore, autoantibody-positive non-RA patients frequently became negative in the second sample (9% for ACPA and 17% for IgM-RF, respectively), whereas autoantibody-negative non-RA patients seldom became positive (Figure 1). When levels in RA patients were the focus, initially ACPA-positive patients showed a nonsignificant median increase from 74 AU/mL (IQR 25 to 252) to 80 AU/mL (IQR 24 to 229), whereas in initially IgM-RF-positive RA patients, median IgM-RF levels decreased (*P *< 0.001) from 94 IU/mL (IQR 51 to 188) to 81 IU/mL (IQR 41 to 178).

### Autoantibody positivity in relation to age

**Figure 2:**
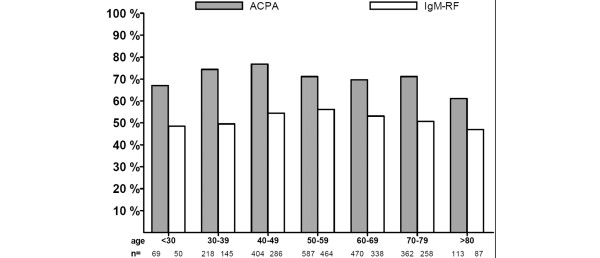
Percentage of rheumatoid arthritis patients with positive antibodies to citrullinated proteins/peptides (ACPA) or IgM rheumatoid factor (IgM-RF) status at the first sample. Patients are grouped according to age.

**Figure 3:**
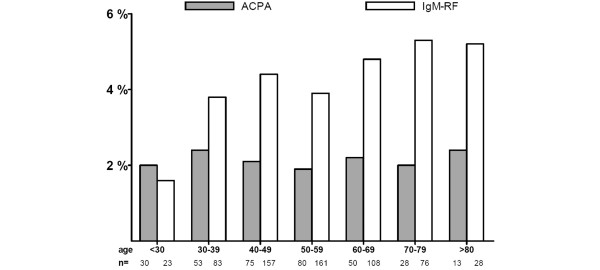
Percentage of non-rheumatoid arthritis patients with positive antibodies to citrullinated proteins/peptides (ACPA) or IgM rheumatoid factor (IgM-RF) status at the first sample. Patients are grouped according to age.

To explore an effect of age on ACPA and IgM-RF positivity, we grouped patients according to their age at the first sample. In RA, ACPA positivity was more frequent than IgM-RF positivity in all age groups. In non-RA, however, the frequency of ACPA positivity was lower than IgM-RF positivity in all age groups except in patients younger than 30 years old. ACPA positivity was similar among the different age groups in RA (Figure 2) as well as non-RA (Figure 3), whereas IgM-RF positivity increased with age in the non-RA group but not in the RA group. The chi-square test for linear trend revealed a significant (*P *< 0.001) linear trend; IgM-RF positivity increased with age in non-RA patients.

### Autoantibody levels in relation to markers of inflammation

In RA patients, a low correlation between autoantibodies and the levels of markers of inflammation (as measured by ESR and CRP) was found. The correlation between IgM-RF levels and the levels of markers of inflammation (ESR: *r *= 0.23; CRP: *r *= 0.21, both *P *< 0.01) was somewhat stronger compared with the correlation of ACPA levels and the levels of markers of inflammation (ESR: *r *= 0.14; CRP: *r *= 0.14, both *P *< 0.01) (Table 1). In a second analysis, the correlation of the changes of levels in time of both antibodies and markers of inflammation – that is, the correlation between (a) the difference between the first and second ACPA/RF levels and (b) the difference between the first and second ESR/CRP levels – was measured. The correlation between change in ACPA levels and change of levels in markers of inflammation (ESR: *r *= 0.16; CRP: *r *= 0.13, both *P *< 0.01) was similar to the correlation at a single time point. For IgM-RF, the correlation of change of levels in time with change of levels in markers of inflammation (ESR: *r *= 0.31; CRP: *r *= 0.28, both *P *< 0.01) was slightly higher compared with the correlation at a single time point. In non-RA patients, no correlation between autoantibody levels and the levels of markers of inflammation was found at a single time point or between changes in ACPA or IgM-RF and markers of inflammation.

**Table 1:** Correlations between autoantibody levels and markers of inflammation

	Erythrocyte sedimentation rate		C-reactive protein	
		
Diagnosis group	ACPA	IgM-RF	ACPA	IgM-RF
RA (n = 1,130)	0.16^a^	0.31^a^	0.13^a^	0.28^a^
Non-RA (n = 1,190)	0.07^b^	0.06	0.02	0.06

^a^*P *< 0.01; ^b^*P *< 0.05. ACPA, antibody to citrullinated proteins/peptides; IgM-RF, IgM rheumatoid factor; RA, rheumatoid arthritis.

## Discussion

Characteristics of ACPA and IgM-RF were studied in a large group of RA and non-RA patients. ACPA status was more stable than IgM-RF status in RA and non-RA patients. ACPA positivity did not increase with age in any group, whereas IgM-RF positivity was stable with age in RA but more frequent in older versus younger non-RA patients. The correlation between autoantibody levels and markers of inflammation was low in RA and absent in non-RA patients.

The results of this study show a low percentage of ACPA seroconversion in both directions compared with IgM-RF. In very early RA, the seroconversion to positivity might occur more frequently [5]. Previous studies of prolonged follow-up of early-arthritis patients seem to show that qualitative changes in ACPA are rare [2,6], although the numbers of patients (n = 96 and 279) were relatively small. In RA, ACPA seroconversion data are available from treatment cohorts, mostly with a follow-up period of less than a year. Most, but not all, studies [16-18] reported a modest decrease in ACPA levels; however, downward seroconversion does not seem to occur. Data on IgM-RF seroconversion in early RA are scarce. In very early RA, a decrease in the percentage of patients positive for IgM-RF was reported [5], and in a study of early RA, 11% of the patients had variable IgM-RF status during 6-year follow-up [19]. In RA, IgM-RF downward seroconversion during anti-TNF treatment has been reported in up to 50% of patients [7,9,20].

With regard to age and autoantibody status, ACPA positivity was stable in both RA and non-RA, whereas IgM-RF positivity increased with age in non-RA, but not in RA. A formal comparison cannot be made, but these results seem to be in line with earlier observations made in healthy older persons. IgM-RF positivity increases with age; in one study, up to 25% of persons more than 85 years old were IgM-RF-positive [10]. In a similar study, ACPA positivity was observed in only 1 out of 300 healthy individuals over 75 years of age [11]. The fact that IgM-RF positivity increases with age in non-RA but not in RA supports the notion that low-affinity RFs associated with infection and older age appear to play an important role in the host response to many infectious organisms and are likely to contribute to host defence. By contrast, high-affinity RFs in RA represent an 'autoimmune humoral signature' that may be independent of age [21]. In a previous study reporting on IgM-RF and ACPA levels in RA, no association was found with age [22].

The present data show modest, but significant, correlations between (changes in) ACPA and IgM-RF levels and the inflammatory indices ESR and CRP. The correlation of changes in autoantibody levels with changes in acute-phase markers was stronger for IgM-RF than for ACPA, which is in line with results seen during anti-TNF treatment [9]. It has been suggested that ACPA is a disease-specific marker because of the stable phenotype, which is confirmed in our results, and IgM-RF acts as a marker of inflammation because it fluctuates with disease activity [9]. Observations of decreasing ACPA or IgM-RF levels were made mainly during treatment with TNF blockers [7].

In this large population, regardless of treatment, ACPA had a low correlation with ESR and CRP and IgM-RF also had a low correlation with ESR and CRP, although the latter correlation was slightly higher both at a single time point and in the course of time. Matsui and colleagues [23] reported similar correlations in a group of RA patients. The slightly stronger correlation of IgM-RF with ESR and CRP supports the notion that IgM-RF acts as a marker of inflammation.

This study has some limitations. Diagnoses were derived from a diagnosis registration system based on the rheumatologist's opinion, used for insurance purposes, and not on standardized criteria. These diagnoses could change over time; the latest diagnosis was used in the study. Furthermore, differences in disease duration and antirheumatic treatment might have influenced these results but this information was unavailable. Anti-TNF treatment may influence ACPA levels; however, it is not likely that this was the cause of the observed change in ACPA positivity since a relatively small number of patients were treated with these agents. Moreover, non-RA patients positive for IgM-RF or ACPA might eventually develop RA since these autoantibodies are present in the preclinical phase [13]. The strength of the study is the large number of observations from routine clinical practice.

## Conclusions

This study shows that, in a large group of rheumatology clinic patients, seroconversion of ACPA in either direction is less frequent than that of IgM-RF. Furthermore, IgM-RF positivity increases with age in non-RA patients, but not in RA patients, whereas ACPA status is stable at different ages. Finally, IgM-RF levels, and to a lesser extent ACPA levels, modestly correlate with markers of the acute-phase response.

## Abbreviations

ACPA: antibody to citrullinated proteins/peptides; AU: arbitrary units; CCP: cyclic citrullinated peptide; CRP: C-reactive protein; ELISA: enzyme-linked immunosorbent assay; ESR: erythrocyte sedimentation rate; IgM-RF: IgM rheumatoid factor; IQR: interquartile range; IU: international units; RA: rheumatoid arthritis; RF: rheumatoid factor; TNF: tumour necrosis factor.

## Competing interests

The authors declare that they have no competing interests.

## Authors' contributions

JU performed analysis and interpretation of data and drafted the manuscript. WHB contributed to the interpretation of data and the drafting of the manuscript. RJvdS collected the data and was involved in the design of the study. BACD helped design the study and draft the manuscript. DvS performed study design, interpretation of data, and drafting of the manuscript. All authors read and approved the final manuscript.
